# Prevalence and under-detection of gambiense human African trypanosomiasis during mass screening sessions in Uganda and Sudan

**DOI:** 10.1186/1756-3305-5-157

**Published:** 2012-08-07

**Authors:** Francesco Checchi, Andrew P Cox, François Chappuis, Gerardo Priotto, Daniel Chandramohan, Daniel T Haydon

**Affiliations:** 1 Faculty of Infectious and Tropical Diseases, London School of Hygiene and Tropical Medicine, Keppel Street, London, WC1E7HT, United Kingdom; 2 Faculty of Epidemiology and Population Health, London School of Hygiene and Tropical Medicine, Keppel Street, London, WC1E7HT, United Kingdom; 3 Médecins Sans Frontières, 78 rue de Lausanne, 1202, Geneva, Switzerland; 4 Geneva University Hospitals & University of Geneva, 14 rue Gabrielle-Perret-Gentil 6, 1211, Geneva, Switzerland; 5 Epicentre, 8Rue Saint-Sabin, 75011, Paris, France; 6 College of Medical, Veterinary and Life Sciences, University of Glasgow, Glasgow, G12 8QQ, United Kingdom

**Keywords:** Trypanosomiasis, Gambiense, Sleeping sickness, Case detection, Screening, Coverage, Prevalence, Uganda, Sudan, Mathematical model

## Abstract

**Background:**

Active case detection through mass community screening is a major control strategy against human African trypanosomiasis (HAT, sleeping sickness) caused by *T. brucei gambiense*. However, its impact can be limited by incomplete attendance at screening sessions (screening coverage) and diagnostic inaccuracy.

**Methods:**

We developed a model-based approach to estimate the true prevalence and the fraction of cases detected during mass screening, based on observed prevalence, and adjusting for incomplete screening coverage and inaccuracy of diagnostic algorithms for screening, confirmation and HAT stage classification. We applied the model to data from three Médecins Sans Frontières projects in Uganda (Adjumani, Arua-Yumbe) and Southern Sudan (Kiri).

**Results:**

We analysed 604 screening sessions, targeting about 710 000 people. Cases were about twice as likely to attend screening as non-cases, with no apparent difference by stage. Past incidence, population size and repeat screening rounds were strongly associated with observed prevalence. The estimated true prevalence was 0.46% to 0.90% in Kiri depending on the analysis approach, compared to an observed prevalence of 0.45%; 0.59% to 0.87% in Adjumani, compared to 0.92%; and 0.18% to 0.24% in Arua-Yumbe, compared to 0.21%. The true ratio of stage 1 to stage 2 cases was around two-three times higher than that observed, due to stage misclassification. The estimated detected fraction was between 42.2% and 84.0% in Kiri, 52.5% to 79.9% in Adjumani and 59.3% to 88.0% in Arua-Yumbe.

**Conclusions:**

In these well-resourced projects, a moderate to high fraction of cases appeared to be detected through mass screening. True prevalence differed little from observed prevalence for monitoring purposes. We discuss some limitations to our model that illustrate several difficulties of estimating the unseen burden of neglected tropical diseases.

## Background

Human African trypanosomiasis (HAT, sleeping sickness) due to *Trypanosoma brucei gambiense* is a neglected, tsetse-fly borne parasitic disease that affects mainly remote and crisis-affected populations of sub-Saharan Africa [1]. Disease begins in a mildly symptomatic, haemo-lymphatic stage (stage 1) and within about 1–2 years progresses to the meningo-encephalitic stage 2, which is fatal unless treated and can leave sequelae [2,3].

Active case detection has been a mainstay intervention to control HAT since the 1920s [4]. It consists of cross-sectional mass screenings, whereby entire communities (usually villages or urban neighbourhoods) are targeted for testing. The screening test is usually the Card Agglutination Test for Trypanosomiasis (CATT), though palpation of lymph nodes in the neck is also often performed (enlarged lymph nodes are a prominent sign of HAT). The confirmation and staging components of the complex diagnostic algorithm [5] are carried out either on site or at a fixed HAT treatment centre, depending on proximity and ease of patient transport. Staging and treatment are often done at the treatment centre, but stage 1 cases are increasingly treated at the community level.

Active case detection prevents disease progression to stage 2 through early treatment irrespective of symptoms; reduces mortality of stage 2 cases; decreases transmission intensity by reducing the infectious pool (humans are thought to be the main ecological reservoir [1]); creates community awareness; and generates an estimate of infection prevalence, the key indicator of HAT burden. Mass screening is empirically associated with reduction in transmission in various settings [6-8], and its decline in the post-colonial era is heavily implicated in the resurgence of HAT in the 1980s and 1990s [9-11].

Active case detection may be indispensible for HAT elimination [6,12]. However, attendance at screening sessions is often low, and diagnostic sensitivity is imperfect [13], limiting its impact. Furthermore, false positives due to imperfect specificity confound prevalence estimates. Here, we use modelling to estimate the fraction of cases detected during mass screening (henceforth referred to as the *detected fraction*) and the true infection prevalence based on data from three Médecins Sans Frontières (MSF) projects in Uganda and Southern Sudan. Estimates of the detected fraction and true prevalence are critical for evaluating the true impact of control programmes and measuring the unseen burden of this neglected tropical disease.

## Methods

### Data sources

We assembled aggregate data from screening sessions conducted in the Kiri (Kajo-Keji county, Southern Sudan), Adjumani and Arua-Yumbe (north west Uganda) MSF projects, previously described [14-17]. Data include village population size (estimated through census by home visitors), numbers screened and cases detected by stage. We excluded sessions that yielded zero prevalence in villages where no cases were detected throughout the project duration. The study was approved by the Ethics Committee of the London School of Hygiene and Tropical Medicine.

### Conceptual framework

Model states and parameters are listed in Table 1. Let *screening coverage* c be the number of people screened divided by the total village population N; *detected fraction* the number of truly positive stage 1 or stage 2 cases detected (S_1,TP_, S_2,TP_) out of all cases prevalent (S_1_, S_2_); and *observed prevalence* the number of cases diagnosed (including false positives) in either stage (S_1,TP_ + S_1,FP_, S_2,TP_ + S_2,FP_), divided by the number of people screened (cN).

**Table 1:** Model parameters

**Parameter**	**Symbol**	**Values**		**Source/Notes**
Village population size	N	Variable		Data
Screening coverage (%)	c	Variable		Data
Relative probability of attending screening (cases versus non-cases)	ρ	Project	Estimate (95% percentiles)	Prediction of step 1 of model. Random values for each iteration sampled from squared deviance distributions of ρ estimates.
		Kiri	1.6 (0.7-12.8)	
		Adjumani	2.5 (1.2-36.6)	
		Arua-Yumbe	1.9 (0.9-4.0)	
Probability that the next person screened is S_1_ or S_2_	p_S1_, p_S2_	from 0 to 1		Updated after each i^th^ person screened. See Equations 4 and 5.
Ratio of observed prevalence at coverage c to observed prevalence at coverage = 100%.	β_c_	Computed for various values of c, and for each MSF project as a whole.		Data and model predictions. See Equation 1 and text.
Diagnostic accuracy		Algorithm	Mode (range)	Random values sampled from the likelihood distributions generated by Checchi *et al*. [13] based on a probabilistic decision model (one random value generated for each iteration). Values for the new Kiri algorithm apply to all screenings conducted since March 2005 (n = 17).
Diagnostic sensitivity in stage 1 (%)	σ_1_	Kiri (old)	98.0 (83.1-99.5)	
		Kiri (new)	57.4 (41.2-78.2)	
		Adjumani	97.9 (74.1-99.2)	
		Arua-Yumbe	96.5 (74.6-98.8)	
Diagnostic sensitivity in stage 2 (%)	σ_2_	Kiri (old)	98.0 (83.5-99.6)	
		Kiri (new)	67.5 (53.6-84.0)	
		Adjumani	97.5 (75.1-99.4)	
		Arua-Yumbe	97.7 (75.0-99.3)	
Diagnostic specificity (%)	φ	Kiri (old)	100.0 (99.8- 100.0)	
		Kiri (new)	100.0 (99.95-100.0)	
		Adjumani	100.0 (99.8-100.0)	
		Arua-Yumbe	100.0 (99.8-100.0)	
Probability of being correctly classified into stage 1 (%)	σ*_1_	Kiri (old)	67.7 (38.5-86.8)	
		Kiri (new)	66.0 (39.0-87.2)	
		Adjumani	70.4 (39.1-88.6)	
		Arua-Yumbe	66.1 (39.2-88.5)	
Probability of being correctly classified into stage 2 (%)	σ*_2_	Kiri (old)	94.7 (82.1-98.6)	
		Kiri (new)	95.1 (81.4-98.4)	
		Adjumani	94.0 (78.7-98.2)	
		Arua-Yumbe	93.1 (78.7-98.2)	
Probability that a false positive case will be classified into stage 1 (%)	ω	Kiri (old)	0.0	Based on the algorithms used in these projects, false positives can only be classified as stage 2 [13].
		Kiri (new)	0.0	
		Adjumani	0.0	
		Arua-Yumbe	0.0	
Binary dummy variables	δ_[…]_	0 or 1		Denote occurrence of event in a given individual.

We hypothesized that the relative probability ρ of attending screening during a session is higher for cases than for non-cases. Accordingly, as screening coverage decreases, the selection bias favouring cases should increase, yielding a higher observed prevalence at coverage c (for c < 1), compared to the prevalence measurable if c = 1. We can thus define a coverage-dependent ratio of observed prevalence for any screening coverage < 1, compared to observed prevalence when everyone is screened:

(1) $$\beta_{c}=\frac{{\left[\frac{S_{obs,c}}{cN}\right]}_{c<1}}{{\left[\frac{S_{obs,c}}{cN}\right]}_{c=1}}$$

Under this hypothesis, β_c_ should increase exponentially as screening coverage decreases.

**Figure 1:**
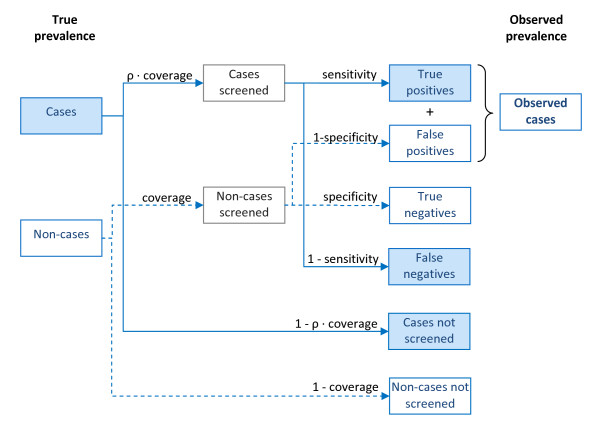
Illustration of the relationship between true and observed prevalence during mass screening.

In addition, observed prevalence is biased upward by false positive tests (incomplete diagnostic specificity), and downward by false negatives (incomplete sensitivity), while the number of stage 1 and stage 2 cases is biased by stage misclassification (Figure 1).

In this paper we develop a static, stochastic mathematical model to predict the relationship between observed prevalence and true prevalence given a specific relative probability ρ of attending a screening session among cases compared to non-cases, which is a parameter we can estimate from field data. This model enabled us to estimate true prevalence and therefore the detected fraction. The different steps in the implementation of the model are outlined in Table 2, and described below.

**Table 2:** Steps in the implementation of the model

	**Step 1**	**Step 2**
**Purpose**	Estimate ρ (relative probability of attending screening among cases versus non-cases)	Estimate the true prevalence and the detected fraction
**Geographical resolution**	Each MSF project	Each screening session (results then totalled over each project)
**Model inputs**	Project-specific diagnostic accuracy parameters	Diagnostic accuracy parameters
	N = 10 000, S_1_ = Uniform [1–50] and S_2_ = Uniform [1–50] (hypothetical values)	Observed N, c, S_1,obs_ and S_2,obs_ for the screening session
	Observed β_c_ (ratio of observed prevalence at coverage c to observed prevalence at coverage = 100%) for four coverage strata (5-24%, 25-44%, 45-64% and 65-84%)	ρ values estimated in Step 1 for each MSF project, sampled from their deviance distribution
	Observed c values sampled from within each coverage stratum and for each project	Various candidate sets of S_1_ and S_2_ (true prevalent cases)
	Various candidate ρ values	
**Model predicted outputs**	β_c_ for the same coverage strata (5-24%, 25-44%, 45-64% and 65-84%)	Number of observed cases (S_1,pred_ and S_2,pred_)
		Number of true positive cases among those observed (S_1,TP,pred_ and S_2,TP,pred_)
**Number of iterations**	10 000 for each project and for each candidate ρ value	10 000 for each screening session and for each candidate set of S_1_ and S_2_
**Fitting procedure**	Predictions fitted against observed β_c_ for the same coverage strata.	Predictions fitted against actual observed cases in screening session (S_1,obs_ and S_2,obs_).
	Observed β_c_ estimated based on a statistical model of field data.	S_1_ and S_2_ candidate sets resulting in best-fitting S_1,pred_ and S_2,pred_ adopted as maximum likelihood estimates of true prevalence. Joint likelihood distribution informs confidence intervals.
	Candidate ρ value resulting in best-fitting β_c_ adopted as point estimate of ρ. Confidence interval based on squared deviance distribution.	

### Description of the mathematical model

The model predicts the number of stage 1 and stage 2 observed cases (S_1,obs_ and S_2,obs_) and the true cases among these (S_1,TP_ and S_2,TP_), based on a set of input parameters, including village population N, true number of prevalent cases S_1_ and S_2_, screening coverage c, relative risk of attending screening among cases versus non-cases ρ, and accuracy (sensitivity, specificity, probabilities of correct stage 1 and 2 classification) of the diagnostic algorithm, as estimated in previous work [13].

Because the number of prevalent cases in a village is often very small and in order to incorporate uncertainty in several parameters, the model was implemented stochastically. Accordingly, individuals in the population have a given probability of experiencing certain events (e.g. attending screening, being detected if positive); chance determines whether the event occurs. The stochastic variation is then examined over a large number of iterations of the model: best estimates and confidence intervals are generated from the distribution of predicted values. Furthermore, during each iteration fresh random values of certain parameters (e.g. diagnostic accuracy) are drawn from their distributions.

### Cases and non-cases screened

The model firstly predicts the number of cases and non-cases screened. If coverage = 1, everyone is screened. If coverage < 1, the situation is akin to sampling without replacement, with sample size = people screened (cN). The probabilities that the i^th^ person screened will be a stage 1 case, stage 2 case or non-case are the product of ρ and the relative proportions of each type of patient in the remaining unscreened population, which change and thus must be updated after each person is screened. Accordingly, the number of cases predicted to be screened over the entire screening session is computed as follows:

(2) $$S_{1,sc,pred}=\sum_{i=1}^{cN}\delta_{1,sc,i},where\;\delta_{1,sc,i}=\{\begin{array}{c} 1,\\ 0, \end{array}\begin{array}{c} Uniform\left[0,1\right]\leq p_{S1,i}\\ Uniform\left[0,1\right]>p_{S1,i} \end{array}$$

(3) $$S_{2,sc,pred}=\sum_{i=1}^{cN}\delta_{2,sc,i},where\;\delta_{2,sc,i}=\{\begin{array}{c} 1,\\ 0, \end{array}\begin{array}{c} Uniform\left[0,1\right]\leq p_{S2,i}\\ Uniform\left[0,1\right]>p_{S2,i} \end{array}$$

In the above equations, random numbers between 0 and 1 are sampled from a uniform distribution to determine whether an event occurs. The probabilities that the next person screened is a stage 1 or stage 2 case are, respectively:

(4) $$p_{S1,i}=\frac{\rho \left(S_{1}-\sum_{j=1}^{i-1}\delta_{1,sc,j}\right)}{\rho \left(S_{1}-\sum_{j=1}^{i-1}\delta_{1,sc,j}\right)+\rho \left(S_{2}-\sum_{j=1}^{i-1}\delta_{2,sc,j}\right)+N-\left(i-1\right)-\sum_{j=1}^{i-1}\delta_{1,sc,j}-\sum_{j=1}^{i-1}\delta_{2,sc,j}}$$

(5) $$p_{S2,i}=\frac{\rho \left(S_{2}-\sum_{j=1}^{i-1}\delta_{2,sc,j}\right)}{\rho \left(S_{1}-\sum_{j=1}^{i-1}\delta_{1,sc,j}\right)+\rho \left(S_{2}-\sum_{j=1}^{i-1}\delta_{2,sc,j}\right)+N-\left(i-1\right)-\sum_{j=1}^{i-1}\delta_{1,sc,j}-\sum_{j=1}^{i-1}\delta_{2,sc,j}}$$

The number of predicted non-cases screened is the total sample cN minus cases screened:

(6) $$H_{sc,pred}=cN-S_{1,sc,pred}-S_{2,sc,pred}$$

In cases where c > 1 (as can occur if people from surrounding villages also attend the screening session), we assumed that the entire village population was screened, i.e. c = 1 for the village in question; additional persons screened from outside the village are ignored in the model, as they do not contribute to the prevalence pool (and thus the detected fraction) within the village in question. MSF datasets specify the origin of cases detected and only cases from the village screened were considered in our analysis. However, when computing observed prevalence, all persons screened (including those from outside the village) were considered in the denominator, as MSF data do not contain the origin of persons screened. In both Uganda and Sudan projects, observed prevalence was also calculated in this way.

### True cases detected

The number of true cases detected among those screened is given by the binomial probability of detection conditional on being screened (diagnostic sensitivity σ), applied to each case screened:

(7) $$S_{1,TP,pred}=Bin\left(S_{1,sc,pred},\sigma_{1}\right)$$

(8) $$S_{2,TP,pred}=Bin\left(S_{2,sc,pred},\sigma_{2}\right)$$

However, some cases detected are misclassified in the wrong stage:

(9) $$S_{1,TP,mis,pred}=Bin\left(S_{1,TP,pred},1-\sigma_{1}^{*}\right)$$

(10) $$S_{2,TP,mis,pred}=Bin\left(S_{2,TP,pred},1-\sigma_{2}^{*}\right)$$

### False positive cases

Out of non-cases screened, some are classified as false positives due to imperfect specificity:

(11) $$S_{FP,pred}=Bin\left(H_{sc,pred},1-\varphi \right)$$

For completeness, we note that some false positives may be classified as stage 1, based on the relative proportion ω of stage 1 s among all false positives, which is highly dependent on the diagnostic algorithm being used:

(12) $$S_{1,FP,pred}=Bin\left(S_{FP,pred},\omega \right)$$

All other false positives are classified as stage 2:

(13) $$S_{2,FP,pred}=S_{FP,pred}-S_{1,FP,pred}$$

In practice, ω was estimated at zero in the MSF projects we analysed [13].

### Predicted observed prevalence

The predicted numbers of cases observed include true and false positives, with some stage misclassification:

(14) $$S_{1,obs,pred}=S_{1,TP,pred}-S_{1,TP,mis,pred}+S_{2,TP,mis,pred}+S_{1,FP,pred}$$

(15) $$S_{2,obs,pred}=S_{2,TP,pred}-S_{2,TP,mis,pred}+S_{1,TP,mis,pred}+S_{2,FP,pred}$$

(16) $$S_{obs,pred}=S_{1,obs,pred}+S_{2,obs,pred}$$

The model's predictions can be plugged into Equation 1 so as to predict β_c_ for any screening coverage level, compared to 100% coverage.

### Step 1: Estimation of the relative probability of attending screening (ρ)

#### Estimation of observed to true prevalence ratio (β_c_) based on field data

We estimated the actual β_c_ within each MSF project and for four screening coverage strata (5-24%, 25-44%, 45-64% and 65-84%), compared to coverage 85-115% as the reference stratum (while this reference stratum should theoretically consist only of screening sessions with c = 100%, in practice very few screening sessions achieved exactly this coverage, and we therefore adopted a wider range assuming that it was practically equivalent to 100%). We estimated β_c_ based on screening data and a statistical model of the association between screening coverage and observed prevalence.

As observed prevalence distributions featured an excess of zeroes and were over-dispersed, a hurdle model [18,19] was used to estimate β_c_, consisting of (i) a first complementary log-log binomial component that models the probability of a non-zero prevalence, and (ii) a second negative binomial component (offset by the natural log of the number of people screened) that models the probability of a given discrete number of cases, conditional on prevalence being non-zero (i.e. on the first “hurdle” of zero having been crossed). This model provided a good fit to the data (results not shown).

In addition to screening coverage, all potential confounding variables available from the data (screening round [first versus subsequent], village population size, observed incidence rate in the six months before the mass screening, and project) were included in the hurdle model. Coefficient standard errors were adjusted for clustering due to repeated screening sessions within individual villages (to do this, "village" was set as the cluster variable).

So as to verify whether ρ differs in stage 2 versus stage 1 cases, we also stratified the hurdle model by stage, and modelled the association between screening coverage and the proportion of stage 2 diagnoses using an alternative group logit regression. Both these analyses (omitted for brevity) suggested no significant difference in β_c_ according to stage; we thus assumed that ρ is equal for stage 1 and stage 2.

#### Estimation of ρ for each MSF project

We implemented the stochastic model described above to predict β_c_ for various coverage values and for each MSF project, as a function of different values of ρ. For each candidate value of ρ in a large plausible range, we examined the distribution of β_c_ generated from 10 000 runs of the stochastic model, and adopted the value of ρ that generated predicted values of β_c_ that best fit those estimated for each site from the available data, i.e. the hurdle model. The value of ρ yielding the best fitting value of β_c_ was selected by minimizing the squared deviation of the predicted β_c_ compared to the actual β_c_, with actual values sampled from the uncertainty distribution provided by the coefficients of the hurdle model (Table 1). The model was run using the diagnostic accuracy parameters specific to each project, sampled from their uncertainty distributions as computed in prior work, and input values of N = 10 000, S_1_ = Uniform [1–50] and S_2_ = Uniform [1–50] (the results were insensitive to input values of N, S_1_ and S_2_). The coverage values at which we predicted β_c_ were also randomly selected from the distribution of screening session coverage values falling within each of the above coverage strata (5-24%, 25-44%, 45-64% and 65-84%).

### Step 2: Estimation of the number of true prevalent cases

We next inputted into the model, for each screening session, the project-specific ρ estimates derived above, sampled from their uncertainty distribution; the actual values of N, c and diagnostic accuracy specific to the session; and candidate sets of S_1_ and S_2_ values (from 0 to N). For each screening session, we evaluated each set of S_1_ and S_2_ values over 10 000 iterations, by computing how frequently the set of values yielded perfect predictions of observed prevalence, i.e.

(17) $$S_{1,obs,pred}=S_{1,obs,data}\;AND\;S_{2,obs,pred}=S_{2,obs,data}$$

For each iteration that yielded a perfect fit, we also recorded the predicted true cases detected S_1,TP,pred_ and S_2,TP,pred_ if they did not exceed the total cases observed $\left(S_{1,TP,pred}\leq S_{1,obs,pred}\;AND\;S_{2,TP,pred}\leq S_{2,obs,pred}\right)$, and those among these that were classified in the correct stage (S_1, TP,pred_ – S_1,TP,mis,pred_ and S_2,TP,pred_ – S_2,TP,mis,pred_). The set of S_1_ and S_2_ most frequently fitting the data was adopted as the best estimate for that screening session. 95% confidence intervals were computed by the method of profiles applied to a two-dimensional joint likelihood distribution [20].

Best estimates and uncertainty bounds for each project as a whole were computed by two alternative analysis approaches: (i) summing the best-fitting values of of S_1_ and S_2_ or S_1,TP_ and S_2,TP_ for each screening session over the project as a whole (no uncertainty bounds could be computed for this approach); and (ii) a bootstrapping routine, whereby we repeatedly sampled from the joint likelihood distributions of S_1_ and S_2_ or S_1,TP_ and S_2,TP_ for each screening session, totalled the randomly sampled values over all sessions in the project, and computed the median and 95% percentile interval of the resulting distribution of random project totals.

S_TP_/S is the detected fraction. We could not find a straightforward way to compute uncertainty bounds around this estimate, as it includes error from several sources arising from different statistical processes. However, we present alternative best estimates of detected fraction using either of the above estimation approaches.

## Results

### Description of mass screening data

#### Screening output

Altogether, 819 mass screening sessions took place in the three projects over the periods covered by the datasets used in this study. However, population data were missing for 203 sessions; 10 yielded zero prevalence in villages that also reported no cases throughout the project duration; and two had a coverage <5% and were assumed to be data entry errors. This left 604 sessions for the present analysis, performed in 246 villages (Table 3).

**Table 3:** Screening coverage of screening sessions included in the analysis, by project

**Coverage stratum (%)**	**Kiri, Sudan (n = 142)**	**Adjumani, Uganda (n = 320)**	**Arua-Yumbe, Uganda (n = 142)**
5-14	1 (0.7)	13 (4.1)	2 (1.4)
15-24	9 (6.3)	26 (8.1)	3 (2.1)
25-34	5 (3.5)	34 (10.6)	5 (3.5)
35-44	13 (9.2)	38 (11.9)	14 (9.9)
45-54	9 (6.3)	49 (15.3)	16 (11.3)
55-64	7 (4.9)	42 (13.1)	15 (10.6)
65-74	8 (5.6)	40 (12.5)	18 (12.7)
75-84	6 (4.2)	38 (11.9)	22 (15.5)
85-94	7 (4.9)	14 (4.4)	23 (16.2)
95-104	4 (2.8)	12 (3.8)	6 (4.2)
105-199	31 (21.8)	12 (3.8)	16 (11.3)
≥200	42 (29.6)	2 (0.6)	2 (1.4)
Mean coverage% (IQR†)	192.9 (51.7-231.0)	58.7 (37.4-74.0)	75.3 (52.5-89.5)
Mean coverage% (IQR†) considering any coverage > 100% as = 100%	77.9 (52.4-100.0)	55.8 (37.6-73.9)	70.6 (52.7-89.3)

†Inter-quartile range.

Screening coverage was highest in Kiri, where about half of screening sessions reported a coverage > 100%, suggesting people from neighbouring communities may have attended (Table 3). Overall, 714 898 people were targeted for screening (with 472 015 actually screened): 56 590 (49 551) in Kiri, 300 406 (158 954) in Adjumani, and 364 902 (263 510) in Arua-Yumbe. Cases diagnosed were 221 (114 in stage 1 or 51.6%) in Kiri, 1419 (692, 48.8%) in Adjumani, and 570 (327, 57.4%) in Arua-Yumbe.

#### Exploration of factors associated with observed prevalence

A hurdle model of factors associated with observed prevalence combining data from all projects (Table 4) suggested weak evidence of a trend in the association between screening coverage and occurrence of non-zero prevalence (log-log component): sessions with coverage <15% were about one third as likely to yield any HAT cases than sessions with coverage around 100%. The probability of non-zero prevalence also increased with village population size and previous observed incidence rate, but was lower in repeat screening rounds.

**Table 4:** Hurdle model exploring factors associated with observed HAT prevalence (all projects combined)

**Variable**	**Number of observations (number with non-zero prevalence)**	**Log-log component: probability of non-zero prevalence**		**Negative-binomial component: prevalence conditional on prevalence being non-zero**		
		**Probability ratio (adjusted)**	**95%CI**	**Prevalence ratio (adjusted)**		**95%CI**
**Screening coverage (%)**						
5-14	16 (5)	0.28†	0.09-0.89	3.39†	**β**_**c**_	1.25-9.21
15-24	38 (19)	0.52	0.25-1.09	2.83		1.58-5.04
25-34	44 (31)	0.78	0.37-1.63	1.77		0.95-3.27
35-44	65 (37)	0.64	0.32-1.28	1.49		0.79-2.81
45-54	74 (46)	0.74	0.37-1.49	1.49		0.85-2.64
55-64	64 (46)	1.18	0.60-2.35	1.47		0.78-2.76
65-74	66 (47)	1.08	0.54-2.17	1.25		0.70-2.24
75-84	66 (46)	0.89	0.44-1.82	1.12		0.62-2.03
85-94	44 (27)	0.79	0.37-1.66	1.19		0.61-2.35
95-104	22 (16)	1	[reference]	1		[reference]
105-199	59 (33)	0.95	0.45-2.00	0.69		0.36-1.32
≥200	46 (25)	1.61	0.65-3.99	0.34		0.15-0.78
**Screening round**						
first round	246 (176)	1	[reference]	1		[reference]
subsequent rounds	358 (202)	0.56	0.44-0.71	0.58		0.47-0.72
**Village population size**						
<100	38 (12)	1†	[reference]	1†		[reference]
100-499	141 (71)	1.98	0.91-4.26	0.50		0.26-0.94
500-999	166 (111)	3.07	1.31-7.20	0.34		0.16-0.71
≥1000	259 (184)	3.84	1.60-9.19	0.23		0.11-0.49
**Observed incidence rate in the past 6 months** (cases per 1000 person-months)						
0.00	239 (100)	1	[reference]	1†		[reference]
0.01-0.99	263 (201)	2.38	1.80-3.15	1.45		1.20-1.77
1.00-4.99	86 (63)	3.04	2.11-4.37	3.40		2.47-4.68
≥5.00	16 (14)	6.08	3.15-11.73	6.16		3.84-9.87
**Project**						
Adjumani	320 (215)	1	[reference]	1		[reference]
Arua-Yumbe	142 (104)	0.77	0.54-1.10	0.40		0.27-0.59
Kiri	142 (59)	0.65	0.41-1.05	1.02		0.70-1.48
		p (goodness of fit): <0.0001		p (goodness of fit): <0.0001		

† Test for trend p < 0.001.

Among screenings that yielded non-zero prevalence (negative binomial component), there was also evidence of a trend in the association of screening coverage and prevalence, with β_c_ increasing as a function of decreasing coverage, as hypothesized. Prevalence increased with previous incidence, but repeat screening rounds were associated with lower prevalence. Unlike in the log-log component, prevalence decreased with increasing population size (see Discussion). There was no evidence of interactions in either model component (data not shown).

### Estimates of the detected fraction

#### Estimated relative risk ρ of attending screening

**Figure 2:**
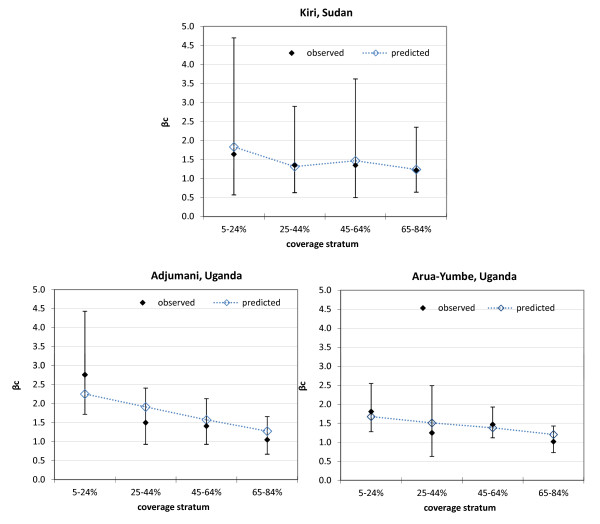
**Predicted versus observed β**_**c**_**(ratio of observed prevalence at coverage c to observed prevalence at coverage = 100%) values, by project, using the best estimate of ρ (relative probability of attending screening among cases versus non-cases).** Vertical bars indicate 95% confidence intervals.

Table 5 shows adjusted estimates of β_c_ based on a hurdle model of field data for each project, used in further steps of the analysis to β_c_. The fit of estimated ρ values was good (Figure 2). The best estimates of ρ were 1.6 (95%CI 0.7-12.8) for Kiri, 2.5 (1.2-36.6) for Adjumani and 1.9 (0.9-4.0) for Arua-Yumbe, suggesting a consistent pattern across sites. These ρ estimates yielded β_c_ values that provided a good fit to the β_c_ values estimated from field data.

**Table 5:** **Adjusted estimates of β**_**c**_**(ratio of observed prevalence at coverage c to observed prevalence at coverage = 100%) for each project, by screening coverage stratum**

**Project**	**Screening coverage stratum (%)**									
	**5-24**		**25-44**		**45-64**		**65-84**		**85-115 (ref.)**	
	**n†**	**β**_**c**_	**n**	**β**_**c**_	**n**	**β**_**c**_	**n**	**β**_**c**_	**n**	**β**_**c**_
Kiri	10	1.64 (0.57-4.70)	18	1.35 (0.63-2.90)	16	1.35 (0.50-3.62)	14	1.22 (0.64-2.35)	17	1 [ref.]
Adjumani	39	2.76 (1.72-4.43)	72	1.50 (0.93-2.41)	91	1.41 (0.93-2.13)	78	1.05 (0.67-1.66)	29	1 [ref.]
Arua-Yumbe	5	1.81 (1.28-2.55)	19	1.25 (0.63-2.49)	31	1.47 (1.12-1.93)	40	1.02 (0.73-1.43)	34	1 [ref.]

†Number in category.

Quantities in parentheses indicate 95% confidence intervals.

#### Estimated true prevalence and detected fraction

The estimated true prevalence using the best-fitting estimates from each session (approach i) was very similar to that observed (Table 6). True prevalence using bootstrapping estimates (approach ii) was almost equal to that observed in Adjumani and Arua-Yumbe, but was about double the observed in Kiri, though still below 1% in absolute terms; the proportion of stage 1 cases was estimated to be higher in reality than that observed, as expected due to the adjustment for stage misclassification, and the fact that most false positives would have been diagnosed as stage 2 (Table 6): observed stage-specific prevalence differed from the true prevalence accordingly.

**Table 6:** Estimated true number of cases and prevalence, by stage, project and overall

**Project**	**Estimated number of cases (95% confidence interval)**			**Prevalence in% (95% confidence interval)**	
	**Observed**	**True cases among observed**	**True cases overall**	**Observed†**	**True‡**
**Kiri**					
stage 1	114	135, 143 (127–158)	177, 315 (255–388)	0.23	0.31, 0.56 (0.45-0.69)
stage 2	107	86, 71 (55–86)	86, 189 (145–257)	0.22	0.15, 0.33 (0.26-0.45)
Total	221	221, 214 (207–219)	263, 507 (429–608)	0.45	0.46, 0.90 (0.76-1.07)
**Adjumani**					
stage 1	692	868, 913 (863–963)	1129, 1628 (1485–1775)	0.44	0.38, 0.54 (0.49-0.59)
stage 2	727	551, 463 (410–513)	648, 993 (872–1128)	0.46	0.22, 0.33 (0.29-0.38)
Total	1419	1419, 1375 (1360–1389)	1777, 2618 (2436–2811)	0.90	0.59, 0.87 (0.81-0.94)
**Arua-Yumbe**					
stage 1	327	404, 392 (366–417)	495, 624 (564–693)	0.12	0.14, 0.17 (0.15-0.19)
stage 2	243	166, 135 (109–162)	153, 262 (214–321)	0.09	0.04, 0.07 (0.06-0.09)
Total	570	570, 527 (510–540)	648, 888 (816–974)	0.21	0.18, 0.24 (0.22-0.27)

†Observed cases divided by the total population actually screened. ‡Estimated cases divided by the total population targeted for screening.

Estimated figures indicate, respectively, sum of best-fitting values for each screening session, median of bootstrapping replicate samples (95% percentile of bootstrapping samples).

Overall, the estimated detected fraction was relatively high everywhere using analysis approach i, i.e. taking the best-fitting estimates from each screening session (84.0% [221/263] in Kiri, 79.9% [1419/1777] in Adjumani and 88.0% [570/648] in Arua-Yumbe), but much lower (42.2% [214/507] in Kiri, 52.5% [1375/2618] in Adjumani and 59.3% [527/888] in Arua-Yumbe) using approach ii, i.e. taking median estimates from bootstrapping (see Discussion). When considering only cases detected and correctly staged, these percentages declined to 68.1% (179/263), 60.4% (1074/1777) and 61.9% (401/648) for approach i, and 33.1% (168/507), 39.9% (1045/2617) and 47.4% (421/888) for approach ii.

## Discussion

This study outlines a potential method to estimate the extent of under-detection and the true infection burden of gambiense HAT, based only on observed data. Because of the extent of uncertainty as regards model parameters, estimates of detected fraction are quite imprecise, but suggest that between 20-50% of prevalent cases were not detected in the screening sessions analysed. There appears to be no appreciable difference between observed and true prevalence. However, adjustment for incomplete specificity and stage misclassification suggests a higher ratio of stage 1 to stage 2 than that observed by programmes.

### Interpretation of findings

#### Internal validity of findings

The hurdle model is internally consistent: with the exception of population size (see below), associations of explanatory variables and prevalence in the log-log component are mirrored in the negative-binomial component.

Furthermore, the log-log component supports the hypothesis of ρ > 1. If ρ = 1, the probability of a village featuring a non-zero observed prevalence should be linearly proportional to screening coverage. However, this probability is higher than expected based on coverage alone, consistent with self-selection of cases even at low coverage.

While increasing village population size was associated with a higher probability of non-zero prevalence, prevalence among non-zero screenings appeared to decrease with higher population. This apparently inconsistent finding may be explained as follows: (i) in fact, the probability of non-zero prevalence increases less than proportionately with increasing population size, meaning that, on a per capita basis, it is lower in large villages than small ones; (ii) in smaller communities, there may be a greater risk of chance extinction of transmission, and thus a greater frequency of zero prevalence, all else being equal; (iii) if cases are present in a small village, their very small number, not divisible below discrete units, affects the prevalence calculation (e.g. if two villages A and B both have one prevalent case, but A’s population is 100 and B’s 1000, the prevalence will be ten times higher in A); (iv) larger communities are usually administrative and economic centres, and attract infected migrants from rural areas; (v) village population size may not reflect the actual denominator at risk: it is likely that only a fraction of the population has a livelihood-dependent exposure to tsetse [21,22], and that this fraction may be smaller in larger, less rural communities where many people are engaged in trade or services: in other words, when considering the true population at risk, denominators might be more comparable across differently sized villages than it appears.

#### Under-detection

Overall, this study estimates that about 20-50% of prevalent cases potentially detectable fell through the net of active case detection, and that about a fourth of cases detected were not classified in the correct stage (however, most misclassification would be from stage 1 to stage 2, which would still guarantee effective treatment). Our model did not incorporate the final step of treatment, as our question concerned case detection specifically; furthermore, the MSF projects used a variety of regimens, including second-line regimens for patients with treatment failure. In national programmes without strong funding and technical support, screening coverage could be lower, and our findings thus reflect an optimistic scenario. In the Democratic Republic of Congo (DRC), the estimated detected fraction (including treatment) was <50% in most scenarios, and between 30% and 65% attended and were correctly diagnosed [23]. Screening coverage was 22-98% in other DRC sites (average 70-80%) [7,23], 47-93% in Equatorial Guinea [24], and 70-94% in Angola [25].

In the colonial era, HAT active case detection was successful due to largely coercive measures. Few recent studies discussing the barriers to and facilitators of screening attendance have been published. In the Republic of Congo, villagers reported that biomedical medicine was the main remedy against HAT, and did not trust traditional remedies [26]. In the DRC, communities’ knowledge of HAT and its control was very good, but concern with drug toxicity and the stigma of public HAT diagnosis were prominent barriers [27]. Both studies found that cost of treatment was a barrier to service uptake; while MSF projects offered free testing and treatment, patients and families face transport costs, income lost, etc. In both Congo [28] and DRC, stage 2 HAT was often associated with sorcery, especially when the case was fatal: however, there was no evidence that this kept patients from seeking care. In the Ugandan sites we analysed, traditional healers were often a recourse, and working with these providers and communities was suggested as a way to improve screening attendance [29].

#### Other findings

In communities where a non-zero incidence was observed in the six months prior to the mass screening, there was a doubled probability of finding at least one case during active screening. Furthermore, past incidence was associated with observed prevalence.

There was no evidence that cases in stage 2 have a greater probability of attending mass screening than those in stage 1. This observation is somewhat unexpected: stage 2 cases, being more symptomatic, might be expected to have a greater probability of attending screenings. This finding, however, may not apply to passive case detection. Furthermore, early stage 2 cases may in fact be less prone to present with systemic symptoms like fever, pruritus or arthralgia than stage 1 cases [30].

#### Programmatic implications

While the uncertainty around the estimates of detected fraction (see below) hampers meaningful interpretation, it is clear that a considerable proportion of HAT infections remain undetected even in a well-resourced active case finding context. These cases would then go on to seed renewed epidemics once mass screening is scaled down, and, where no passive case detection is available, would probably die. Long-term control of HAT through mass screening thus probably requires very high screening coverage, underscoring the need for programmes to work closely with communities to ensure high acceptance and uptake and identify and address barriers to screening attendance. This is also justifiable from an economic standpoint, given that the costs of mass screening are mainly fixed (e.g. transport, human resources, information campaigns, programme overheads) rather than variable (i.e. per person screened).

This study suggests that, for purposes of assessing HAT burden and monitoring trends, calculating the observed prevalence based on detected cases and the number of people screened provides a reasonable approximation to the true prevalence. Furthermore, programmes should continue to use the observed incidence in different communities (as computed based on passive case detection, where available) as a guide for deciding where to focus mass screening efforts.

### Study limitations

Estimates were subject to considerable uncertainty, which hampers interpretation of the key findings on detected fraction. The striking differences according to analysis approach are due to the very skewed likelihood distributions arising from the fitting procedures (data not shown): reporting the mode (best-fitting values) or median of these distributions changes the inference considerably. For completeness, we have chosen to report both, and suggest that reality lies somewhere in between. Furthermore, the model does not adequately deal with screening sessions featuring zero observed prevalence (37% of sessions analysed). If screening coverage is < 100%, various possible sets of S_1_ and S_2_ values could result in S_1,obs_ = 0 and S_2,obs_ = 0; in most scenarios, however, the set [S_1_ = 0, S_2_ = 0], i.e. zero prevalence, will by default yield the best fits and will thus be adopted as the best estimate, potentially resulting in a systematic underestimation of true prevalence in very low transmission villages (and overestimation of the detected fraction) if analysis approach i is used. Approach ii is less affected by this bias.

For screening sessions with coverage > 100%, the model relies on an assumption that the entire population of the village was screened, and that any other persons screened come from neighbouring villages. While this occurred rarely in Adjumani and Arua-Yumbe, in Kiri about half of screening sessions attracted a population greater than that of the village; results for Kiri should thus be considered somewhat less robust.

The association between coverage and observed prevalence was adjusted for all available confounding variables, but these (screening round, population size, project, past incidence) were few, and additional hidden confounding may be present: villages with low coverage may differ systematically from high-coverage ones in other key determinants of prevalence, such as exposure to vectors; low coverage might also be a proxy for remoteness and low security, which could be associated with higher prevalence.

## Conclusions

The fraction of HAT cases detected during active screening may be relatively high in well-resourced control programmes, providing a considerable immediate public health benefit. However, the minority of cases that remain undetected may play a critical epidemiological role in sustaining transmission.

The limitations of this study illustrate multiple difficulties in estimating the unseen burden of neglected tropical diseases in settings with low access to health care and limited availability of data. Our modelling approach may be useful for improved HAT burden estimation and programme evaluation, but needs to be improved.

Determinants of under-detection should also be researched further using both quantitative and qualitative tools, so as to maximise the future impact of this control strategy.

## Competing interests

We declare that we have no competing interests.

## Authors’ contributions

FChe designed the study, carried out analyses, interpreted findings and wrote the manuscript. AC carried out analyses, interpreted findings and co-wrote the manuscript. FCha and GP collected data, interpreted findings and co-wrote the manuscript. DC interpreted findings and co-wrote the manuscript. DH designed the study, interpreted findings and co-wrote the manuscript. All authors read and approved the final manuscript.
